# Congenital heart disease-associated pulmonary dysplasia and its underlying mechanisms

**DOI:** 10.1152/ajplung.00195.2022

**Published:** 2022-12-06

**Authors:** De-Bao Li, Xiu-Xia Xu, Yu-Qing Hu, Qing Cui, Ying-Ying Xiao, Si-Juan Sun, Li-Jun Chen, Lin-Cai Ye, Qi Sun

**Affiliations:** ^1^Department of Thoracic and Cardiovascular Surgery, Shanghai Children’s Medical Center, School of Medicine, Shanghai Jiao Tong University, Shanghai, People’s Republic of China; ^2^Department of Thoracic and Cardiovascular Surgery, Shanghai Institute for Pediatric Congenital Heart Disease, Institute of Pediatric Translational Medicine, Shanghai Children’s Medical Center, Shanghai School of Medicine, Shanghai Jiao Tong University, Shanghai, People’s Republic of China; ^3^Department of Cardiology, Shanghai Children’s Medical Center, School of Medicine, Shanghai Jiao Tong University, Shanghai, People’s Republic of China; ^4^Department of Radiology, Huangpu Branch, Shanghai Ninth People’s Hospital, School of Medicine, Shanghai Jiao Tong University, Shanghai, People’s Republic of China; ^5^Department of Pediatric Intensive Care Unit, Shanghai Children’s Medical Center, School of Medicine, Shanghai Jiao Tong University, Shanghai, People’s Republic of China

**Keywords:** alveoli, congenital heart disease, human, lung, pulmonary dysplasia

## Abstract

Clinical observation indicates that exercise capacity, an important determinant of survival in patients with congenital heart disease (CHD), is most decreased in children with reduced pulmonary blood flow (RPF). However, the underlying mechanism remains unclear. Here, we obtained human RPF lung samples from children with tetralogy of Fallot as well as piglet and rat RPF lung samples from animals with pulmonary artery banding surgery. We observed impaired alveolarization and vascularization, the main characteristics of pulmonary dysplasia, in the lungs of RPF infants, piglets, and rats. RPF caused smaller lungs, cyanosis, and body weight loss in neonatal rats and reduced the number of alveolar type 2 cells. RNA sequencing demonstrated that RPF induced the downregulation of metabolism and migration, a key biological process of late alveolar development, and the upregulation of immune response, which was confirmed by flow cytometry and cytokine detection. In addition, the immunosuppressant cyclosporine A rescued pulmonary dysplasia and increased the expression of the Wnt signaling pathway, which is the driver of postnatal lung development. We concluded that RPF results in pulmonary dysplasia, which may account for the reduced exercise capacity of patients with CHD with RPF. The underlying mechanism is associated with immune response activation, and immunosuppressants have a therapeutic effect in CHD-associated pulmonary dysplasia.

## Graphical abstract

**Figure:**
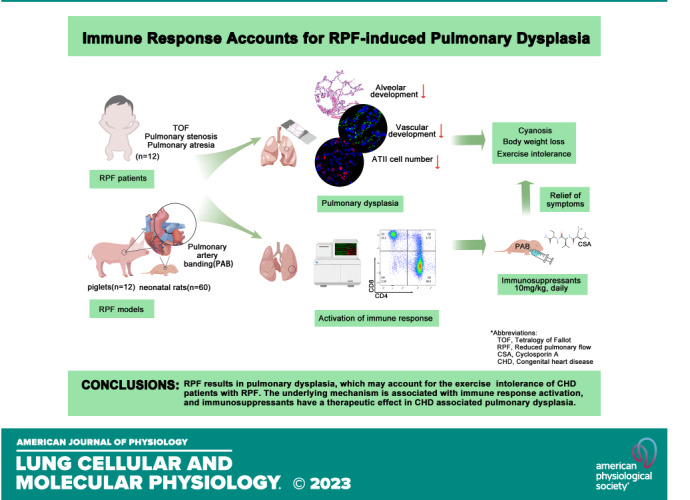

## INTRODUCTION

Although the life expectancy of children with congenital heart disease (CHD) has greatly improved with the advances that have been made in cardiac surgical and perioperative technology, their exercise capacity remains significantly lower than that of healthy controls (1, 2). Exercise capacity is an important determinant of survival in pediatric patients with CHD and correlates with their quality of life (3–5). Recent clinical observations have demonstrated that exercise capacity is more significantly reduced in patients with CHD with reduced pulmonary blood flow (RPF) than those with normal or increased pulmonary blood flow (6). In light of this, the underlying mechanisms by which RPF impairs exercise capacity may yield insights to improve the long-term quality of life of pediatric patients with CHD.

A person’s exercise capacity is largely dependent on their respiratory and cardiovascular performance and the profound, functional, and anatomical interplay between both (7–9). The respiratory performance of children is determined by vascular and alveolar development in the lungs, where 95% of alveoli form during the first 7 years of life in humans (10, 11). Fetal studies demonstrated that pulmonary blood flow modulates the development of fetal pulmonary parenchyma and vessels (12, 13), yet little is known about whether pulmonary blood flow modulates postnatal lung development. Moreover, although a clinical study of adult patients with congenital pulmonary valve stenosis, which leads to RPF, revealed that these patients had smaller lungs than healthy controls (11), whether the smaller lungs found in such patients are caused by RPF remains unclear.

Incomplete pulmonary development is the main characteristic of bronchopulmonary dysplasia (BPD), one of the most common complications of premature infants that affects ∼40% of extremely preterm newborns (born at <28 wk of gestation; 14, 15). Epidemiological research has shown that 58% of extremely preterm newborns have pulmonary hypertension, which leads to RPF (16, 17). However, the current most popular hyperoxia injury-induced BPD animal model does not mimic the symptoms of RPF in preterm infants (18, 19), and targets derived from these models have limited efficacy in clinical treatments (20, 21). If RPF is one of the contributors to BPD, RPF animal models may serve as alternative pulmonary dysplasia models for preterm newborns.

Therefore, in this study, we first selected lung tissues from patients with tetralogy of Fallot to demonstrate whether RPF caused pulmonary dysplasia with similar characteristics to those of BPD. Then, we performed pulmonary artery (PA) banding (PAB) surgery on piglets and rats to further demonstrate whether RPF results in pulmonary dysplasia in large animals and rodents. Third, RNA sequencing (RNA-seq) was performed on lung tissues from PAB rats to uncover the possible pathways regulating pulmonary dysplasia. Finally, pathway regulators were tested to check whether they have a treatment effect on the symptoms caused by RPF.

## METHODS

All of the RNA-seq data were deposited in the gene expression omnibus (GEO) database (https://www.ncbi.nlm.nih.gov/geo) under the accession No. GSE201522.

### Ethics Statement

All of the study procedures conformed to the principles outlined in the Declaration of Helsinki and were approved by the Animal Welfare and Human Studies Committee at Shanghai Children’s Medical Center (Institutional Review Board Approval No. SCMCIRB-Y2020094). For human experiments, written informed consent was obtained from the parents of the children. Rats were euthanized by cervical dislocation after being anesthetized with pentobarbitone (50 mg/kg ip injection). Piglets were euthanized using an overdose of pentobarbitone (150 mg/kg iv injection).

### Infant RPF Lung Samples

A total of 12 tiny lung tissue samples (5 mm × 5 mm × 5 mm) were collected from patients during routine cardiothoracic surgery in Shanghai Children’s Medical Center, Shanghai, PR China, from January 2021 to December 2021, including six patients suffering from RPF and another six patients with normal pulmonary blood flow. All of the procedures performed were done in accordance with the Animal Welfare and Human Studies Committee at the Shanghai Children’s Medical Center. The patients’ clinical information is presented in Table 1.

**Table 1.** Patients’ clinical information

Group	Diagnosis	Age, Months	PA Velocity, m/s	PA Diameter, cm	PA Flow, L/s
RPF	TOF	5	4	0.7	0.015386
	TOF	6	4.49	0.41	0.05925
	TOF	4	4.04	0.56	0.09946
	TOF	5	5.91	0.45	0.09395
	TOF	5	4	0.55	0.09499
	TOF	4	4.63	0.41	0.0611
Mean		4.8	4.52	0.51	0.09377
SD		0.7	0.74	0.11	0.03436
Control	COA	1	1.2	0.9	0.11606
	COA	10	1.66	1.21	0.25907
	COA	1	1.25	0.8	0.1266
	COA	6	1.1	1.24	0.13277
	COA	3	0.8	0.93	0.1592
	COA	4	0.6	1.26	0.13849
Mean		4.17	1.10	1.05	0.15537
SD		3.43	0.37	0.20	0.05279
*P* value		0.6519	<0.0001**	0.0002**	0.03761*

COA, coarctation of the aorta; PA, pulmonary artery; RPF, reduced pulmonary blood flow; TOF, tetralogy of fallot. *P < 0.05, **P < 0.01.

### Animal RPF Lung Samples

To obtain animal RPF lung samples, we performed PAB surgery on neonatal rats and piglets. The animal experiments in this research were approved by the ethics committee of Shanghai Children’s Medical Center. Timed-pregnant (*embryonic day 18*) wild-type Sprague–Dawley rats (RRID: RGD_70508) were obtained from Xipu’er-bikai Experimental Animal Co., Ltd. (Shanghai, PR China) and were housed two per cage until they gave birth. Piglets were purchased from Jiagan Biological Technology Co., Ltd. (Shanghai, PR China) and were housed one per cage until 2 mo. Animals were fed under the same conditions with free access to food and water and maintained in the 12-/12-h day/night cycle. The pups were divided randomly into PAB and sham groups, the latter of which was subjected to the same operation as the former, except for the banding step. A total of 10 piglets and 60 rat pups (male or female) were enrolled in this study.

### Neonatal PAB Surgery

Neonatal PAB surgery was performed according to our previous publications (22, 23). Briefly, neonatal pups were anesthetized by hypothermia on ice for ∼3 min, then transferred to an ice bed to maintain anesthesia during the operation. The surgery, including sternum clipping, thoracic cavity opening, pericardium incising, PA quantitative constriction, and thoracic cavity closing, was performed under a stereomicroscope in the supine position. The constriction procedure is shown in Fig. 3*B* (and a surgical video is provided in Supplemental Video S1). After their chests were closed, the pups were removed from the ice bed and warmed on a heat plate, then put back with their moms.

The piglet PAB surgery was performed according to a previous publication (24). Briefly, left-side thoracotomy in the fourth or fifth intercostal space was performed under general anesthesia and mechanical ventilation. The PA was exposed through partial pericardiotomy. The banding tapes were placed through the bottom of the main PA (MPA) and then constricted to create a twofold increase in peak velocity of the MPA as measured by transesophageal echocardiography. The chest was subsequently closed following routine procedures, and postoperative analgesia was conducted for 3 days.

### Transthoracic Echocardiography

Transthoracic echocardiography was performed on *postnatal day 14* (*P14*) by a single experienced echocardiologist blindly to verify MPA stenosis and quantify the pulmonary blood flow. Rats were anesthetized with isoflurane (isoflurane/oxygen, 5%) in an induction chamber for 3–5 min, then put into the warming plate in a supine position with a nasal cone. The anesthesia was maintained by the inhalation of 1.5%–2% isoflurane/oxygen through a nasal cone. A Vevo 2100 echocardiography system (Visual Sonics, Toronto, ON, Canada) equipped with a 25-MHz transducer (MS400 Micro-scan Transducer; Visual Sonics) was used to conduct the echocardiography inspection. To assess PA stenosis, the blood flow images in color Doppler mode, mean pressure gradient (MPG), and velocity-time integral (VTI) data in pulsed-wave Doppler mode were acquired from the long-axis view of the MPA. To calculate the pulmonary blood flow, the heart rate (HR), VTI, and aortic root diameter (AoD) from the long-axis view of the aortic outflow tract were obtained. According to continuity law, the pulmonary blood flow was calculated as pulmonary blood flow = (AoD/2)^2^ × π × VTI × HR.

### Morphological Examination and Tissue Preparation

Lung morphometry analysis was conducted at *P14*. After the rats were euthanized, their ribs on both sides along the mid-axillary line were cut to expose the thoracic cavity. The lungs were removed and washed in PBS. The gross morphology of each lung was taken under a Leica M205 FA stereomicroscope (Leica Microsystems, Wetzlar, Germany).

For histological and immunohistological studies, the rats and piglets were euthanized at *P14*. Briefly, the thoracic cavity was exposed, a small incision was made in the left atrium, and the PBS was used to clear the blood in the pulmonary vascular bed by slowly perfusing the PA. The alveoli were fixed and inflated by instilling the tissue fixation fluid through the trachea (0.2 mL/10 g, 5 cmH_2_O). Then, the lung tissues were removed, rinsed in PBS solution, and fixed at room temperature with 4% paraformaldehyde (pH, 7.4) overnight. Afterward, the tissues were dehydrated via an ethanol series, embedded in paraffin, and sliced into 5-µm sections.

For RNA-seq study, the lung tissues were cut into 1–2 cm pieces, snap-frozen in liquid nitrogen, and then stored in a refrigerator at −80°C.

### Immunofluorescence

Immunofluorescence staining of Von Willebrand factor (Vwf, RRID: AB_1960544), CD31, and surfactant protein C (Sftpc), and terminal transferase-mediated dUTP nick end labeling (TUNEL) were performed in this study. For vWF, CD31, and Sftpc staining, the paraffin-embedded lung sections were dewaxed in xylene, hydrated using an alcohol gradient, and subjected to antigen retrieval. After 1 h of blocking using PBS with 7.5% goat serum and 0.5% Trixon X-100, the sections were incubated overnight with primary rabbit anti-vWF (ab6994, dilution, 1:200; Abcam, Cambridge, UK), anti-CD31 (A4900, dilution, 1:100; Abclonal, Wuhan, PR China), and anti-Sftpc (PA5-71680, dilution, 1:50; Thermo Fisher Scientific, Waltham, MA) antibodies at 4°C. The next day, primary antibodies were washed using PBST (PBS with 1% Tween), and the sections were incubated with Alexa Fluor 488 goat anti-rabbit fluorescent secondary antibody (ab150077, dilution, 1:500; Abcam, Cambridge, UK) for 1 h at room temperature. Cell nuclei were stained with DAPI (No. P0131; Beyotime Biotechnology, Shanghai, PR China), and the sections were mounted using an antiquenching resident medium and sealed with nail polish. TUNEL staining was accomplished using a 1-step TUNEL apoptosis assay kit (No. C1089; Beyotime Biotechnology, Shanghai, PR China) according to the manufacturer’s guidance. Briefly, the sections were incubated with 20 μg/mL of protein K for 30 min at room temperature, then hydrated as described earlier. Next, 50 µL of the TUNEL detection solution (consisting of 5 µL of terminal deoxynucleotide transferase enzyme and 45 µL of fluorescein labeling solution) was added to each tissue, and the sections were incubated at 37°C, protected from light for 1 h, and then sealed for observation.

### Alveolarization and Vascularization Assessment

Hematoxylin and eosin (H&E) staining was performed to evaluate alveolarization using an H&E staining kit (Solarbio, Shanghai, PR China) according to routine protocols and was imaged under an optical microscope. The extent of alveolarization was quantified with mean linear intercepts (MLIs; 25, 26) by the ImageJ software (https://imagej.nih.gov/ij; U.S. National Institutes of Health, Bethesda, MD). Briefly, to assess MLI, grids consisting of horizontal and vertical lines were positioned on the image and the total number of times the borderline intersected with the alveoli and the total length of the grids was recorded. MLI was defined as: MLI = (total grid length/total number of intersections) in micrometers (μm). Immunofluorescence staining for alveolar type 2 (AT2) cells marker Sftpc was also performed in this study to evaluate lung growth (27). The results were quantified by average Sftpc intensity. To determine lung vascular density, immunofluorescence staining for endothelial markers vWF and CD31 was performed and imaged under confocal microscopy. The pulmonary vascular density was defined as the average intensity of CD31.

### RNA-Seq and Enrichment Analyses

High-throughput sequencing was performed at *P14* to detect pulmonary differentially expressed genes (DEGs; *n* = 5/group). RNA was first extracted from lung tissue and reverse-transcribed to complementary DNA (cDNA) in M-MuLV reverse transcriptase and DNA polymerase I. AMPure XP beads were used to select cDNA fragments with a length of 370–420 bp and were used for PCR product purification. A library was constructed using the NEBNext Ultra RNA Library Prep Kit for Illumina. Library and RNA quality were assessed with the Agilent 2100 bioanalyzer (Agilent Technologies, Santa Clara, CA). RNA integrity number (RIN) values were all >9.

Clustering was subsequently performed on a cBot cluster-generation system using the TruSeq PE version 3-cBot-HS cluster kit (Illumina, San Diego, CA) according to the manufacturer’s instructions. The Novaseq platform (Illumina) was used for library preparation sequencing.

Raw data in. fastq format were then processed to obtain clean data by removing reads containing N base, adapter, or low-quality information. Q20, Q30, and GC were calculated in the meantime. Sequence reads were aligned to the rat reference genome (assembly rnor_6.0) using HISAT2 version 2.5.0. Novel transcripts prediction was performed by StringTie (v.1.3.3b; 28). Then, the read numbers of each gene were counted using featureCounts version 1.5.0-p3. The expected number of fragments per kilobase of transcript sequence per million base pairs sequenced of each gene was calculated based on the length of the gene and the reads count mapped to said gene. Differential expression analysis of two groups was conducted using the DESeq2 R package (v.1.20.0). To control the false-discovery rate, the adjusted *P* value was calculated using Benjamini and Hochberg’s approach. Adjusted *P* values <0.05 and absolute fold-changes >2 were considered to be statistically significant.

Gene Ontology (GO) and Kyoto Encyclopedia of Genes And Genomes (KEGG) enrichment analyses of DEGs were then implemented using the cluster profile R package. GO terms with corrected *P* values <0.05 were assigned as significantly enriched. KEGG pathways were enriched from DEGs based on the KEGG database (http://www.genome.jp/kegg/).

### Flow Cytometry

Lung digestion was performed to obtain single-cell suspensions for flow cytometry as described elsewhere (29). Briefly, each lung was exposed and perfused with 1 mL of PBS to remove red blood cells, then lavaged and inflated by digestion liquid containing elastase (4 U/mL in PBS, CE5001, Coolaber, Beijing, PR China), dispase II (1 U/mL in PBS, CD4691, Coolaber, Beijing, PR China) DNase (200 μg/mL in water, DNASE70, Sigma, MO), and liberase (5 mg/mL in PBS, 5401020001, Wolcavi, Beijing, PR China) at 37°C for 45 min. The lung was then cut into small pieces and transferred into a 70-μm cell strainer to collect cell suspension. The remaining red blood cells were removed by incubation with red blood cell lysis buffer (00-4333-57, eBiosciences, Waltham, MA) for 5 min. Cells were subsequently washed, resuspended in PBS containing 2% fetal bovine serum three times, and blocked with 2% normal rabbit serum. The cells were subsequently stained with CD3, CD4, CD45, and CD8 antibodies (559975, 550057, 561588, 559976; 1:100; eBiosciences, Waltham, MA) at 4°C for 30 min. After being washed three times, the cells were resuspended and analyzed by flow cytometry (FACSAria Fusion; Becton, Dickinson and Company, Franklin Lakes, NJ). Data were analyzed using the FlowJo software.

### Cell Apoptosis Evaluation

Cell apoptosis was assessed by performing immunofluorescence staining for TUNEL. Images were captured under a confocal microscope, and nuclei costained by the target antibody and DAPI were considered positive. The proportion of positive cells to total cells was used as the quantitative index to evaluate cell apoptosis. Ten randomly selected slides in each tissue sample were selected for positive staining cell counting.

### Western Blotting

Western blotting was performed to detect the expressions of Wnt3a and β-catenin proteins of the sham, RPF, and cyclosporine A (CsA) therapy rat groups at 14 days of age. To extract the total protein, lung tissues were homogenized in radioimmunoprecipitation assay lysis buffer (25 mg/mL) with phenylmethylsulfonyl fluoride (1 mM) and centrifuged at 3,000 *g* at 4°C. The supernatants were subsequently collected for the quantification of protein concentration using a standard bicinchoninic acid protein assay kit (P0012; Beyotime Biotechnology, Shanghai, PR China). Then, a 20-μg protein sample with 8-μL loading buffer was mixed and diluted to 24 μL with ddH_2_O. The mix was loaded for 15% sodium dodecyl sulfate-polyacrylamide gel electrophoresis, 20 μL per lane, and transferred to nitrocellulose membranes electrophoretically. Furthermore, 5% nonfat milk was used for membrane blocking for 1 h at room temperature. Subsequently, the membrane was washed in TBST (150 mM saline and 10 mM Tris with 0.05% Tween-20) three times and incubated with the primary antibodies Wnt3a (ab219412; dilution, 1:1,000; Abcam, Cambridge, UK) and β-catenin (ab32572; dilution, 1:5,000; Abcam, Cambridge, UK) at 4°C overnight. The primary antibody was detected with horseradish peroxidase-conjugated secondary antibody (dilution, 1:5,000; Beyotime Biotechnology, Shanghai, PR China). Blots were scanned using the AmerSham Imager 600. The primary and secondary antibodies were then removed using Stripping buffer (Beyotime Biotechnology, Shanghai, PR China). The membrane was reblocked and restained with β-actin (ab8229; dilution, 1:500; Abcam, Cambridge, UK) following the same procedure and then scanned. The expression levels were quantified using the ImageJ software program.

### Statistical Analysis

Quantitative data are expressed as means ± standard deviation values. Statistical analyses were performed using an unpaired, two-tailed Student’s *t* test or one-way analysis of variance test and Student’s Newman–Keuls for post hoc test if variables were normally distributed; otherwise, the rank-sum test was used for comparison. *P* < 0.05 was considered to be statistically significant. The SAS version 9.2 software (SAS Institute, Cary, NC) was used for all statistical analyses.

## RESULTS

### RPF Causes Pulmonary Dysplasia in Human Infants

As shown in Table 1 and Supplemental Fig. S1, the PA diameter was significantly reduced in the RPF group compared with the control group (0.51 ± 0.11 cm vs. 1.05 ± 0.20 cm, *P* = 0.0002), whereas the PA velocity was significantly increased (4.52 ± 0.74 m/s vs. 1.10 ± 0.37 m/s, *P* < 0.0001). Correspondingly, the PA flow was significantly reduced in the RPF group compared with the control group (0.09377 ± 0.03436 L/s vs. 0.15537 ± 0.05279 L/s, *P* = 0.0376).

**Figure 1.**
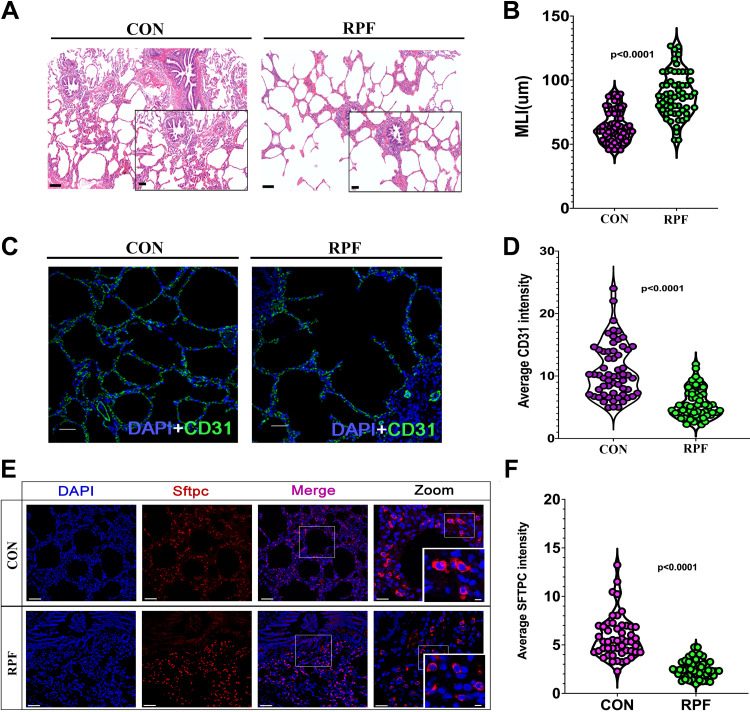
Reduced pulmonary blood flow (RPF) causes pulmonary dysplasia in human infants. *A*: representative hematoxylin and eosin (H&E) staining of lung tissues from RPF and control (CON) lungs. Scale bar: 100 μm in ×10 images, 50 μm in ×20 images. *B*: quantification of mean linear intercept (MLI). *n* = 60 slides from 12 samples. *C*: representative CD31 staining of lung tissues from RPF and CON lungs. CD31 (green), DAPI (blue). Scale bar: 75 μm. *D*: quantification of CD31 intensity. *n* = 60 slides from six samples. *E*: representative AT2 cells from RPF and CON lungs. Surfactant protein C (Sftpc, red), DAPI (blue). *F*: quantification of Sftpc intensity. *n* = 60 slides from 12 samples. Scale bar: 75 μm in ×63 images, 5 μm in zoom images. The scale bars are similar in the following images.

H&E staining showed that MLI, indicating alveolar simplification, was significantly increased in the RPF group compared with the control group (*P* < 0.0001, Fig. 1, *A* and *B*). Immunostaining showed that CD31 intensity, an indicator of blood vessels, was significantly reduced (*P* < 0.0001, Fig. 1, *C* and *D*) in the RPF group compared with the control group. The AT2 cells are of great importance to the alveoli as they not only secrete surfactant proteins to maintain the stability of the pulmonary sac but may also function as stem cells that differentiate to AT1 cells during damage repair (27). Compared with the control group, the expression of Sftpc, a marker of the AT2 cell, was significantly downregulated in the RPF group (*P* < 0.0001, Fig. 1, *E* and *F*). These results suggested that RPF causes impaired alveolarization and vascularization in human infants similar to the condition of infants with BPD.

### RPF Causes Pulmonary Dysplasia in Piglets and Rats

**Figure 2.**
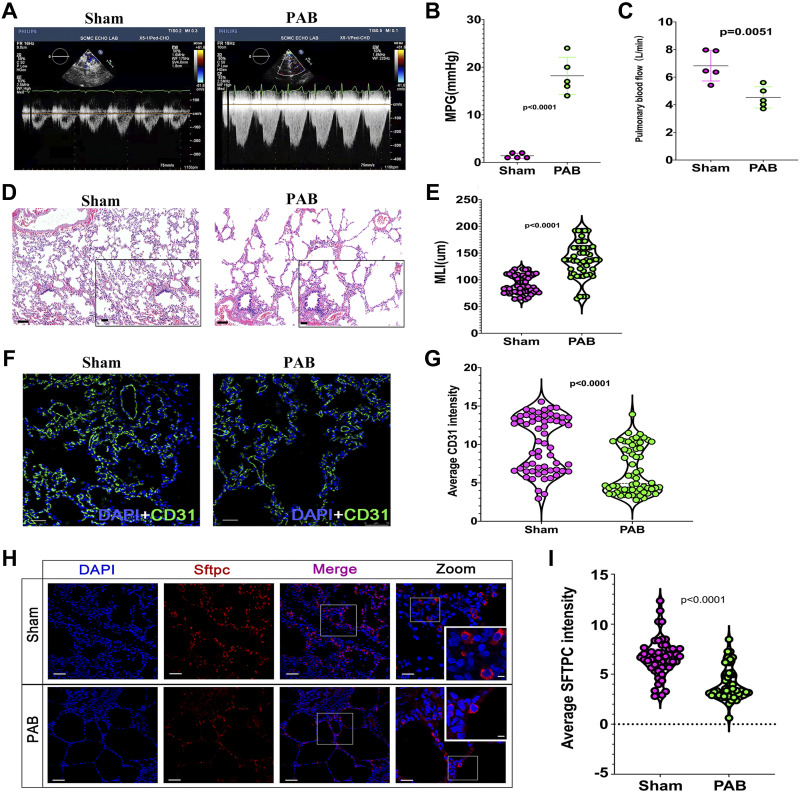
Reduced pulmonary blood flow (RPF) causes pulmonary dysplasia in piglets. *A*: representative echocardiography of pulmonary artery (PA) banding (PAB) and sham piglets. *B*: quantification of the mean pressure gradient (MPG) of PAB and sham piglets. *n* = 5 samples. *C*: quantification of pulmonary blood flow of PAB or sham piglets. *n* = 5 samples. *D*: representative hematoxylin and eosin (H&E) staining of lung tissues from PAB and sham piglets. *E*: quantification of mean linear intercept (MLI). *n* = 50 from 10 samples. *F*: representative CD31 staining of lung tissues from PAB and sham piglets. CD31 (green), DAPI (blue). *G*: quantification of CD31 intensity of lung tissues from PAB and sham piglets. *n* = 50 slides from 10 samples. *H*: representative AT2 cells from PAB and sham piglets. Surfactant protein C (Sftpc, red), DAPI (blue). *I*: quantification of Sftpc intensity. *n* = 50 slides from 10 samples.

To confirm the results obtained from human infants, we performed PAB surgery to create RPF in neonatal piglets and rats. As shown in Fig. 2, *A–C*, PAB significantly increased MPG and caused RPF in piglets. As a result, MLI was significantly increased (*P* < 0.01, Fig. 2, *D* and *E*), whereas CD31 and Sftpc intensity were significantly reduced (*P* < 0.01, Fig. 2, *F–I*) in the lungs from the PAB group compared with the sham group. These results indicated that RPF caused pulmonary dysplasia in neonatal piglets, which is consistent with the findings in human infants (Fig. 1, *A* and *B*).

**Figure 3.**
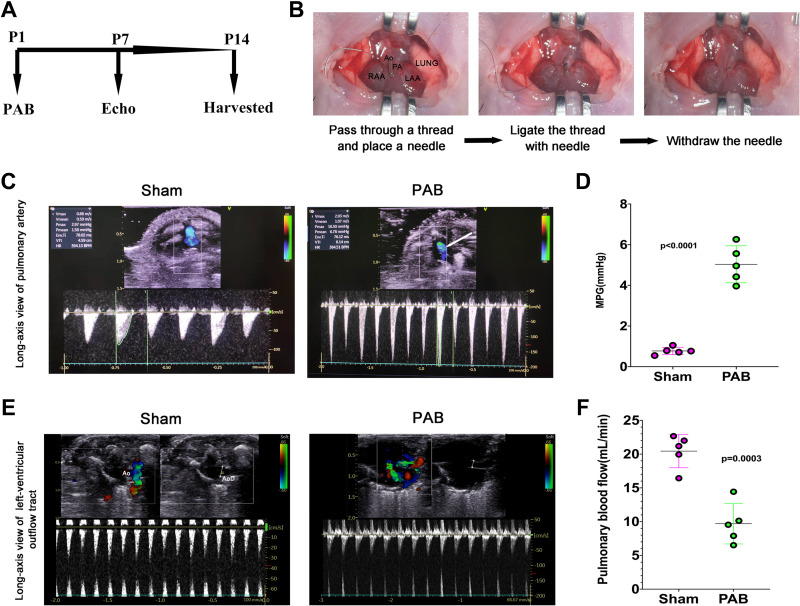
Neonatal rat reduced pulmonary blood flow (RPF) model construction. *A*: rat experimental flowchart. *B*: illustration of the pulmonary artery (PA) banding (PAB) procedure. *C*: representative transthoracic echocardiography of PA. The white arrow represents the narrow, irregular, and colorful blood flow signal, indicating a high-speed blood flow with a stenosis area. *D*: quantification of the mean pressure gradient (MPG) of PAB and sham rats. *n* = 5 samples. *E*: representative transthoracic echocardiography of the left ventricular outflow tract. *F*: quantification of pulmonary blood flow. *n* = 5 samples. Ao, aorta; LAA, left atrial appendage; PA, pulmonary artery; RAA, right atrial appendage; RPA, right pulmonary artery.

**Figure 4.**
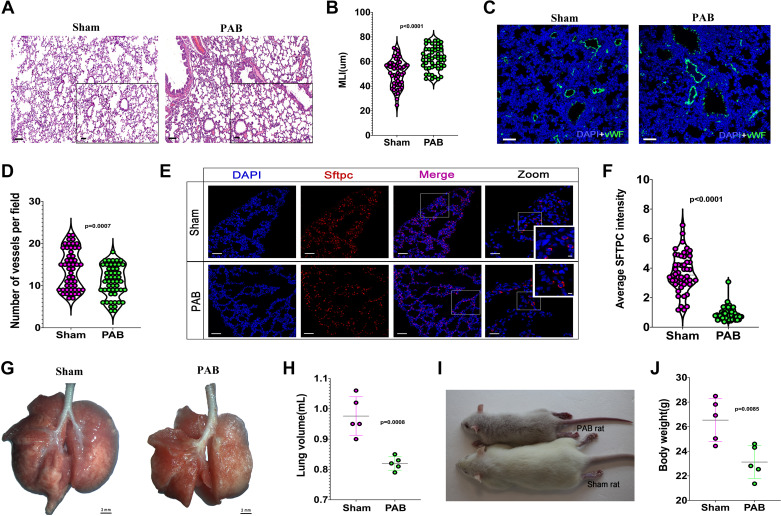
Reduced pulmonary blood flow (RPF) causes pulmonary dysplasia in rats. *A*: representative hematoxylin and eosin (H&E) staining of lung tissues from pulmonary artery (PA) banding (PAB) and sham rats. *B*: quantification of mean linear intercept (MLI). *n* = 50 from five samples. *C*: representative Von Willebrand factor (vWF) staining of lung tissues from PAB and sham rats. vWF (green), DAPI (blue). *D*: quantification of vWF intensity of lung tissues from PAB and sham rats. *n* = 50 from five lungs. *E*: representative alveolar type 2 (AT2) cells from PAB and sham lungs. Surfactant protein C (Sftpc, red), DAPI (blue). *F*: quantification of Sftpc intensity. *n* = 50 slides from five lungs. *G*: representation of whole lungs from PAB and sham rats. *H*: quantification of the lung volume from sham and PAB rats. *n* = 5 lungs. *I*: representative PAB and sham rats. Noted that there is cyanosis and body weight loss in PAB rats. *J*: quantification of body weight of PAB and sham rats. *n* = 5 rats.

As neonatal rats have limited surgical space, creating surgical difficulty, we provided a video (Supplemental Video S1) and illustration of neonatal PAB surgery for researchers to learn the skill (Fig. 3, *A* and *B*). PAB significantly increased PA velocity (Fig. 3, *C* and *D*) and induced RPF in rats (Fig. 3, *E* and *F*). Similar to humans and piglets, RPF also caused incomplete lung development in neonatal rats (Fig. 4, *A–F*). As shown in Fig. 4, *G–J*, RPF led to reduced lung volume and decreased body weight (Fig. 4, *G–J*), which are symptoms also observed in human beings with RPF (30). Besides, cyanosis was also more evident in the RPF rats compared with the sham-operated rats, which indicates the declined exercise tolerance (Fig. 4*I*; 31, 32).

To further assess pulmonary dysplasia, we performed pulmonary function testing at *P14*. As expected, although there were no significant differences in pulmonary resistance, compliance, and elastance between the control and RPF groups, there were some shifts of these parameters as early as *P14* (Supplemental Fig. S2).

### RPF Activates Apoptosis-Induced Inflammation

**Figure 5.**
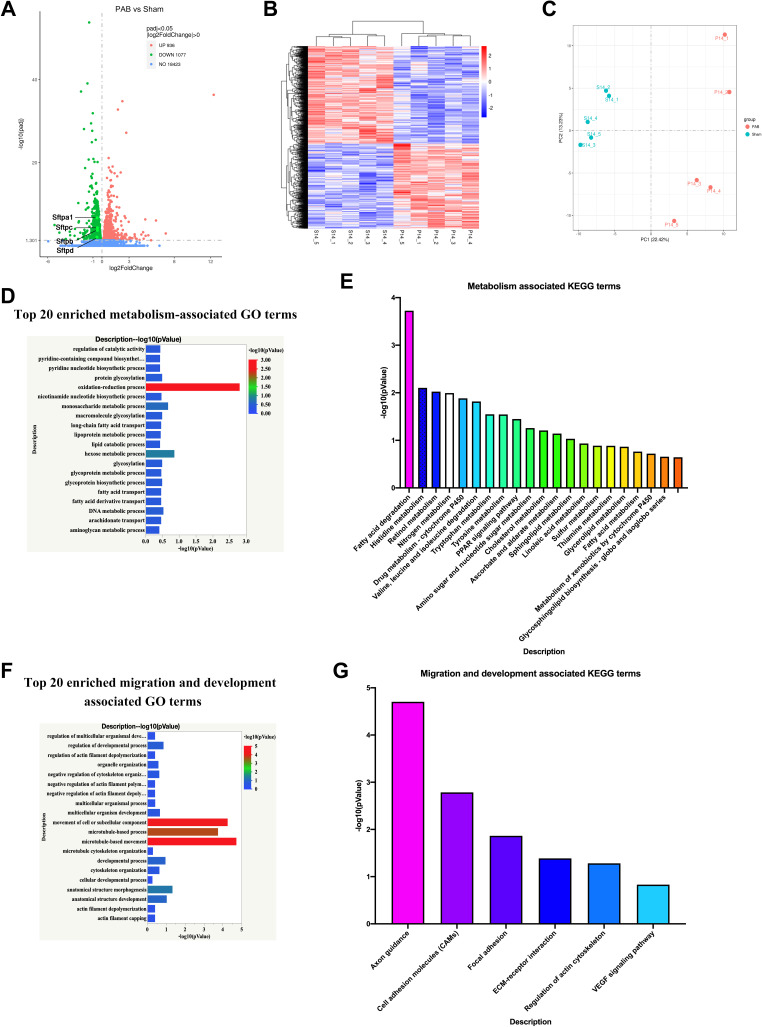
Reduced pulmonary blood flow (RPF) disturbs the late development of the alveolus. *A*: volcano map of the differentially expressed genes. The surfactant proteins (Sftpa, Sftpb, Sftpc, Sftpd) are all on the list of differentially expressed genes (DEGs). *B*: heat map of DEGs. *C*: PCA plots of DEGs. *D*: top 20 enriched metabolism-associated gene ontology (GO) terms. *E*: top 20 enriched metabolism-associated Kyoto Encyclopedia of Genes And Genomes (KEGG) terms. *F*: top 20 enriched migration and development-associated GO terms. *G*: top 20 enriched migration and development-associated KEGG terms.

There were 2,013 DEGs between the sham and PAB groups, among which 936 were upregulated and 1,077 were downregulated (Fig. 5*A*). A cluster analysis based on the heat map revealed a greater similarity of the specimen within the sham or PAB group than among groups (Fig. 5*B*). A PCA plot was used to analyze the variability between- and within groups. As shown in Fig. 5*C*, there was good repeatability in each group and obvious differences across groups. GO and KEGG pathway analysis of downregulation DEGs indicated abundantly enriched terms of cell migration and metabolism, which are the characteristics of late alveolarization (*P9* to *P18*; Fig. 5, *D–G*; 33). Cell migration is required for alveolar morphogenesis, and metabolism is required for the production of alveolar surfactant and homeostasis (33). These results further confirmed that RPF caused pulmonary dysplasia, with similar characteristics to those of BPD.

**Figure 6.**
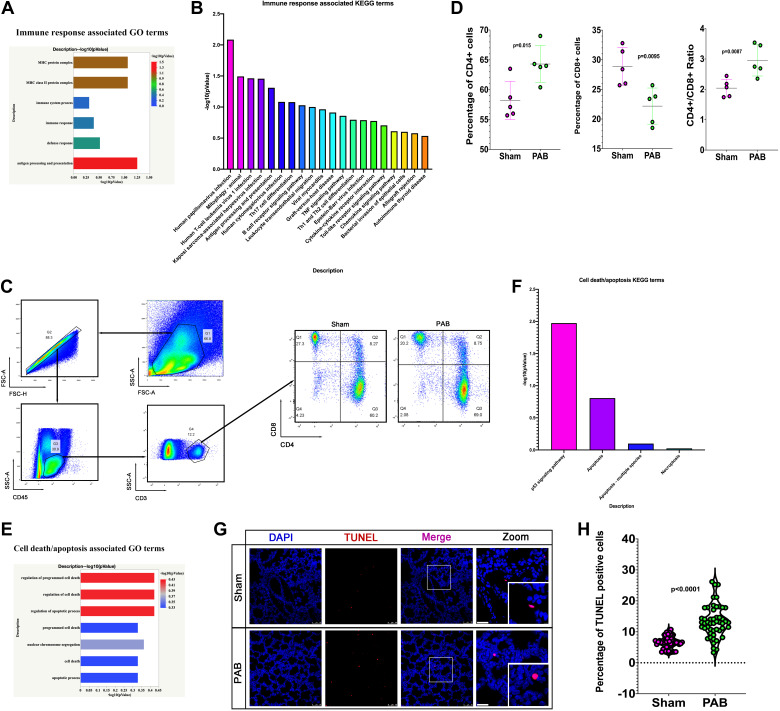
Reduced pulmonary blood flow (RPF) induces apoptosis and inflammation in the lungs. *A*: the most significantly enriched immune response-associated gene ontology (GO) terms. *B*: top 20 enriched immune response-associated Kyoto Encyclopedia of Genes And Genomes (KEGG) terms. *C*: representative flow cytometry plots of CD4+/CD8+ cells from sham and pulmonary artery (PA) banding (PAB) groups. *D*: quantification of the percentage of CD4+, CD8+ cells, and ratio of CD4+/CD8+ cells. *E*: the most significantly enriched GO terms of cell apoptosis/death. *F*: the most significantly enriched KEGG terms of cell apoptosis/death. *G*: representative terminal transferase-mediated dUTP nick end labeling (TUNEL)-positive cells from sham and PAB groups. *H*: quantification of the percentage of TUNEL-positive cells from sham and PAB groups. *n* = 50 slides from five lungs.

GO and KEGG pathway analyses of DEGs also indicated abundantly enriched terms of upregulation of the immune response (Fig. 6, *A* and *B*). Flow cytometry confirmed the upregulation of the immune response, showing that RPF increased CD4+ cells and the ratio of CD4+/CD8 cells (Fig. 6, *C* and *D*). To further confirm the results, we detected the cytokines in the serum and lung tissue lysates, and the results showed that there was a significant increase in inflammatory cytokines in both serum and lung lysates (Supplemental Fig. S3) in the RPF group. These results indicate that RPF activates inflammation in the lungs.

Next, we asked why inflammation was activated by RPF. GO and KEGG pathway analysis of DEGs revealed abundantly enriched terms of cell death/apoptosis (Fig. 6, *E* and *F*). Immunostaining results showed that RPF increased the percentage of TUNEL-positive cells in the lungs (Fig. 6, *G* and *H*), which was consistent with the findings of enrichment analysis. These results indicated that the inflammation in RPF lungs may be induced by cell death/apoptosis. Since inflammation plays a vital role in preterm BPD (2), inflammation may account for the underlying mechanism of pulmonary dysplasia.

### Immunosuppressant CsA Rescues Pulmonary Dysplasia Caused by RPF

**Figure 7.**
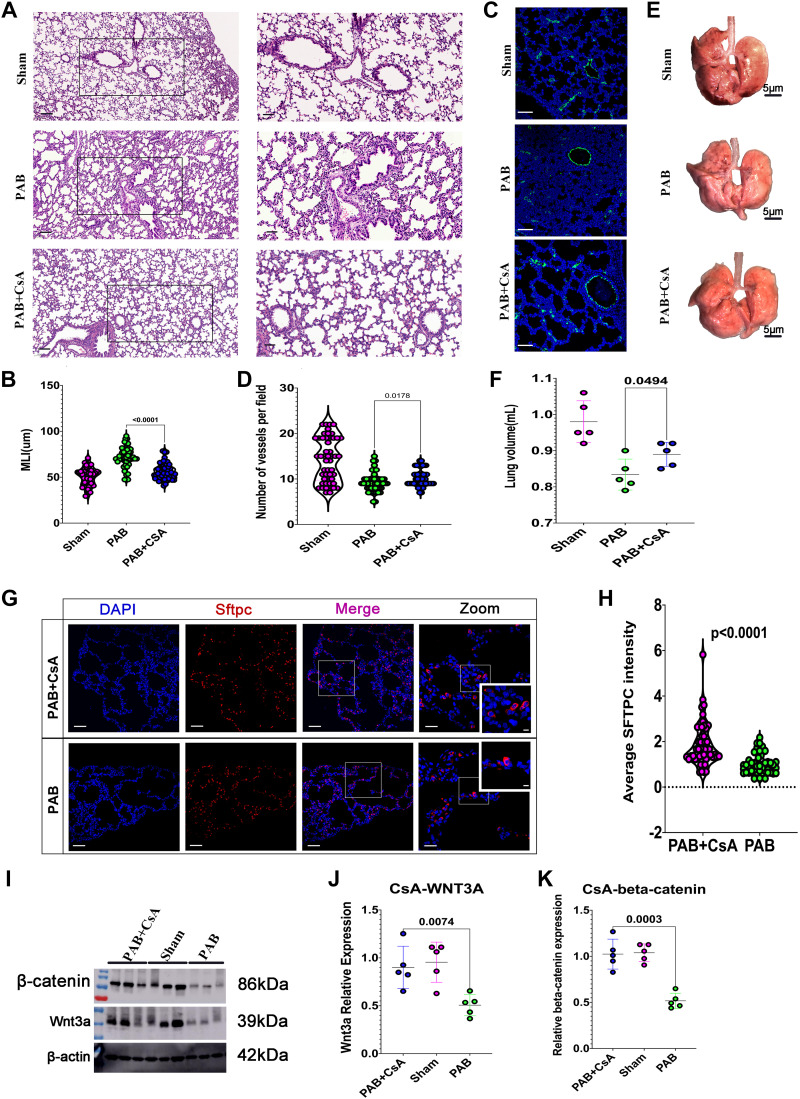
Immunosuppressant cyclosporine A (CsA) rescues pulmonary dysplasia by activating the Wnt signaling pathway. *A*: representative hematoxylin and eosin (H&E) staining of lung tissues from sham, pulmonary artery (PA) banding (PAB), or CsA-treated rats. *B*: quantification of mean linear intercept (MLI). *n* = 50 slides from five lungs. *C*: representative Von Willebrand factor (vWF) staining of lung tissues from sham, PAB, or CsA-treated rats. vWF (green), DAPI (blue). *D*: quantification of vWF intensity of lung tissues. *n* = 50 slides from five lungs. *E*: representation of whole lungs from sham, PAB, or CsA-treated rats. *F*: quantification of the lung volume from sham, PAB, or CsA-treated rats. *n* = 5 lungs. *G*: representative alveolar type 2 (AT2) cells from PAB and CsA-treated lungs. Surfactant protein C (Sftpc, red), DAPI (blue). *H*: quantification of Sftpc intensity. *n* = 50 slides from five lungs. *I*: representative Wnt3a/β-catenin blot from sham, PAB, or CsA-treated rats. *J*: quantification of Wnt3a. *n* = 5 samples. *K*: quantification of β-catenin. *n* = 5 samples.

To further confirm the role of inflammation in pulmonary dysplasia caused by RPF, we treated PAB rats with the immunosuppressant CsA according to a previous publication (34). As shown in Fig. 7, *A–D*, MLI was significantly reduced by CsA therapy, whereas the pulmonary vascular number was significantly elevated after CsA therapy, indicating that CsA rescued RPF-induced alveolar and vascular damage. As a result, RPF-induced smaller lung volume was rescued (Fig. 7, *E* and *F*). In addition, the expression of AT2 cells was significantly elevated in the CsA therapy group (Fig. 7, *G* and *H*). These results confirmed that inflammation plays a critical role in RPF-induced pulmonary dysplasia and suggested that CsA has a therapeutic effect on the symptoms.

### CsA Upregulated the Wnt Signaling Pathway

Recent studies suggest that Wnt signaling pathway drives postnatal alveolar development (35, 36). Since Wnt3a and β-catenin are two of the most important factors in the Wnt signaling pathway, we checked the expression of both Wnt3a and β-catenin to investigate whether the therapeutic effect of CsA was associated with the Wnt signaling pathway. As shown in Fig. 7, *I–K*, RPF significantly downregulated the expressions of Wnt3a and β-catenin compared with that of sham-operated rats, whereas CsA therapy significantly upregulated their expression levels. These results indicated that CsA may improve the exercise tolerance of children with RPF-associated CHD via reactivating the Wnt signaling pathway.

## DISCUSSION

CHD with RPF, encountered in patients with conditions marked by right ventricular outflow obstruction, such as tetralogy of Fallot, pulmonary stenosis/atresia, or pulmonary hypertension, accounts for 13% of all CHD cases (37). Exercise capacity, measured by the maximum oxygen uptake (V̇o_2max_), is lowest in the RPF type of CHD (2) and declines nearly 2% per year in children with CHD (38). Thus, understanding how RPF causes reduced exercise capacity may provide insight into improving the long-term quality of life in children who suffer from CHD.

A clinical trial demonstrated that impaired pulmonary function is an independent predictor of mortality in adult CHD, and pulmonary function should be monitored early in life (2). Reduced lung volume and impaired pulmonary function were observed in patients with CHD with RPF. A study on a swine pulmonary stenosis model demonstrated that early stenting intervention was associated with improved pulmonary development compared with delayed stenting intervention (39).

Considering that exercise capacity is dependent on pulmonary function (2, 3), these results suggest that impaired postnatal pulmonary development may be responsible for the reduced exercise capacity in patients with CHD with RPF. However, the underlying mechanisms by which RPF impairs postnatal pulmonary development have scarcely been investigated. The current study demonstrated for the first time that cell death-/apoptosis-induced immune response is one of the underlying mechanisms accountable for the impaired pulmonary development, suggesting that antiapoptosis or immune response inhibition may be a treatment option to improve exercise capacity in pediatric patients with CHD with RPF. Indeed, current data showed that the immunosuppressant CsA has a therapeutic effect on RPF in CHD, and the underlying mechanism was associated with enforced expression of Wnt, the driver of postnatal pulmonary development.

In addition, we provided an alternative animal model for preterm BPD, which was characterized by impaired alveolarization and vascularization, and inflammation (18). Current preterm BPD animal models include hyperoxia, mechanical ventilation, or direct inflammation-induced pulmonary injury animal models (18), yet none of these mimic RPF in preterm infants. Prospective studies showed that pulmonary hypertension is quite prevalent in preterm infants, especially in those with severe BPD, affecting up to 58% (15, 16). Current animal models of BPD fail to mimic the clinical features of RPF in preterm infants, so the therapeutic targets derived from these models are not ideal (16, 20). An RPF-related pulmonary dysplasia animal model may bring new and effective targets for the treatment of preterm BPD.

It should be noted that patients who have increased pulmonary blood flow, such as those with ventricular septal defects, have also been noted to have abnormal respiratory mechanics (40–42). It has been implicated that there is an association with abnormalities in the lymphatic circulation, which requires further investigation (43).

Another important concern is the form of blood flow in the regulation of lung growth and exercise intolerance. Patients who underwent the Fontan operation, characterized by nonpulsatile flow, are associated with exercise intolerance (44). The underlying mechanisms may be associated with blood flow-mediated production of nitric oxide (NO), an important regulator of vascular bed remodeling and resistance (45). The loops of blood flow form, NO, pulmonary development, and exercise tolerance need to be illustrated in further investigations to understand CHD-associated pulmonary dysplasia.

There are several issues awaiting answers in the current study. For example, by which targets the inflammation/immune response inhibits Wnt signaling pathways was not investigated. Using transgenic animals could solve this problem and may provide specific targets to avoid the side effects of CsA in the treatment of pulmonary dysplasia.

In summary, the current study used human, piglet, and rat RPF lung samples to demonstrate that RPF caused pulmonary dysplasia with similar characteristics to those of BPD. We demonstrated that the underlying mechanism of reduced exercise tolerance in pediatric patients with CHD with RPF may be pulmonary dysplasia. Furthermore, we found that inflammation plays a role in RPF-induced pulmonary dysplasia, and CsA could alleviate the symptoms caused by RPF. In addition, the current study suggested a pulmonary dysplasia animal model as a complement to currently used preterm BPD animal models.

## DATA AVAILABILITY

RNA-seq data were deposited in the Gene Expression Omnibus (GEO) database (https://www.ncbi.nlm.nih.gov/geo) under the accession No. GSE201522. Data will be made available upon reasonable request.

## SUPPLEMENTAL DATA

10.6084/m9.figshare.20296596 Supplemental Fig. S1: https://doi.org/10.6084/m9.figshare.20296596.

10.6084/m9.figshare.21411645 Supplemental Fig. S2: https://doi.org/10.6084/m9.figshare.21411645.

10.6084/m9.figshare.21420324 Supplemental Fig. S3: https://doi.org/10.6084/m9.figshare.21420324.

10.6084/m9.figshare.20296599 Supplemental Video S1: https://doi.org/10.6084/m9.figshare.20296599.

## GRANTS

This work was supported by grants from the Shanghai Natural Science Foundation 22ZR1479900, the National Natural Science Foundation of China Grants 82001835 and 82270314, the Foundation of Pudong Science and Technology Development Grant PKJ2019-Y12, the Science and Technology Innovation Action Plan of Shanghai—Experimental Animal Research Grant 201409005900, the Innovative Research Team of High-Level Local Universities in Shanghai, the National Natural Science Foundation of China (81870238), Shanghai Science and Technology Innovation Project (19411950200), Key Discipline Group Development Fund of Health and Family Planning Commission of Pudong New District (PWZxq2017-14), and Shanghai Key Clinical Specialty (shslczdzk).

## DISCLOSURES

Q. Sun and L.-C. Ye are applying for a patent that covers the use of immunosuppressant CsA in the treatment of pulmonary dysplasia in children who suffer from CHD with RPF (Patent application No. CN202211385332.7). None of the other authors has any conflicts of interest, financial or otherwise, to disclose.

## AUTHOR CONTRIBUTIONS

X.-X.X., L.-C.Y., and Q.S. conceived and designed research; D.-B.L., X.-X.X., Y.-Q.H., Q.C., Y.-Y.X., S.-J.S., L.-J.C., L.-C.Y., and Q.S. performed experiments; D.-B.L., X.-X.X., Y.-Q.H., Q.C., Y.-Y.X., S.-J.S., L.-J.C., L.-C.Y., and Q.S. analyzed data; D.-B.L., X.-X.X., L.-J.C., L.-C.Y., and Q.S. interpreted results of experiments; D.-B.L., L.-C.Y., and Q.S. prepared figures; D.-B.L., L.-C.Y., and Q.S. drafted manuscript; D.-B.L., L.-C.Y., and Q.S. edited and revised manuscript; D.-B.L., X.-X.X., Y.-Q.H., Q.C., Y.-Y.X., S.-J.S., L.-J.C., L.-C.Y., and Q.S. approved final version of manuscript.
